# Comparison of Short-Term Outcomes: Minimally Invasive Thoracotomy Versus Median Sternotomy for Atrial Septal Defect Closure

**DOI:** 10.7759/cureus.77898

**Published:** 2025-01-23

**Authors:** Ata Ullah, Abduz Zaher, Heemal Saha, Sumaiya Mamun, Shahanara Akhter

**Affiliations:** 1 Department of Cardiac Surgery, Bangabandhu Sheikh University, Dhaka, BGD; 2 Institute of Nutrition and Food Science, University of Dhaka, Dhaka, BGD; 3 Department of Cardiac Surgery, Bangabandhu Sheikh Mujib Medical University, Dhaka, BGD; 4 Department of Gynecology, Noakhali 250 Bed General Hospital, Noakhali, BGD

**Keywords:** adult congenital heart disease (achd), asd closure, atrial septal defect, comparing short-term, median sternotomy, minimally invasive thoracotomy

## Abstract

Objective: This study aimed to compare the early outcomes of atrial septal defect (ASD) surgeries performed using minimally invasive (MI) thoracotomy versus conventional median sternotomy (MS).

Materials and methods: Fifty patients with ASD were included in this study conducted at Bangabandhu Sheikh Mujib Medical University, Dhaka. Fifteen patients underwent MI thoracotomy (Group A), while 35 underwent conventional MS (Group B). Eligible patients were over 16 years old with a single ASD, while those requiring additional interventions were excluded. Demographic data, surgical details, and outcomes were analyzed using SPSS Statistics version 26.0 (IBM Corp. Released 2019. IBM SPSS Statistics for Windows, Version 26.0. Armonk, NY: IBM Corp.). Pain levels, complications, and other variables were compared using appropriate statistical tests, with a p-value <0.05 considered statistically significant. The study adhered to ethical guidelines and received institutional approval.

Results: Early outcomes of ASD surgeries were analyzed between the two groups. Group A had a younger mean age (31.77 ± 2.64 years) compared to Group B (36.33 ± 12.61 years), although the difference was not statistically significant (p = 0.624). Both groups had similar body mass index (BMI), ASD size, and gender distribution. Group A experienced significantly longer operation times and lower intraoperative body temperatures (p < 0.001). Postoperative drainage was higher in Group A (p = 0.048). However, Group B required more inotropic support (25.71% vs. 13.33%, p = 0.011) and had more significant pain management needs during the recovery period. No significant differences were observed in other outcomes or complications between the groups.

Conclusions: MI thoracotomy for ASD closure offers several advantages over conventional MS, including reduced pain, faster recovery, and shorter hospital stays. Patients in the MI thoracotomy group experienced fewer complications, including atrial fibrillation, arrhythmias, and atelectasis, with no cases of reoperation for bleeding, neurological complications, wound infections, conversions to sternotomy, or mortality. They also required less analgesia and benefited from superior cosmetic outcomes, making this approach a more favorable option, particularly for younger patients.

## Introduction

Congenital heart disease (CHD) remains the leading cause of major congenital anomalies, representing a significant global health challenge [1]. In 2017, the global prevalence of CHD was estimated at approximately 1.8 cases per 100 live births, with an annual global mortality rate of 261,247 deaths [2]. Among the various forms of CHD, atrial septal defects (ASDs) are the most common congenital heart defects in adults and the second most common in children [3]. ASDs are characterized by significant left-to-right shunting, which leads to progressive complications such as right ventricular volume and pressure overload. If left untreated, these conditions can result in right ventricular failure, irreversible pulmonary hypertension, life-threatening arrhythmias, and paradoxical embolization. Patients with symptomatic ASDs commonly present with atrial arrhythmias, atrial fibrillation, fatigue, dyspnea, and exercise intolerance, all of which significantly impact quality of life. However, many individuals with ASDs remain asymptomatic for long periods, often adapting their lifestyles to manage symptoms [3].

The global birth prevalence of ASD is approximately 2.5 per 1,000 live births, with 25-30% of cases persisting into adulthood [4]. In Bangladesh, ASD is notably prevalent, with Khan et al. (2022) reporting that 25% of children diagnosed with CHD had ASD [5]. The incidence of ASD among live-born CHD patients is estimated at 7.4% [6]. Additionally, Fatema et al. (2008) observed that 26% of babies born with CHD had ASD in a study of 5,668 live births at a hospital [7].

The first successful closure of an ASD was performed in 1953 by Lewis and Taufic (1953), marking a landmark achievement in congenital heart surgery. Since then, various surgical approaches have been developed, including ASD closure using conventional median sternotomy (MS) and minimally invasive (MI) thoracotomy. Both methods demonstrate relatively low mortality and morbidity rates, with conventional MS remaining the standard procedure for ASD closure in pediatric and adult patients [8-10]. While relatively straightforward compared to other complex heart surgeries, conventional MS can leave a conspicuous scar and may be associated with complications [9,11].

In response to these concerns, many surgical centers have adopted MI thoracotomy for ASD closure. This approach offers advantages such as improved postoperative sternum stability, reduced risk of deep infections, enhanced respiratory function and mobility, and minimized bleeding [12]. Additional benefits include smaller skin incisions, reduced pain, faster recovery, quicker resumption of daily activities, and shorter hospital and ICU stays [13].

Evidence shows that MI surgery offers good long-term results. Studies indicate that MI surgery leads to less long-term pain after the procedure, higher patient satisfaction, and fewer severe complications like chest instability and infections compared to conventional MS [14-16]. Additionally, MI surgery helps patients recover better, reduces the chances of hospital readmissions, and improves their quality of life in the long run [17]. These benefits have made MI surgery an increasingly popular choice, especially for patients seeking quicker recovery and minimal disruption to their daily routines.

However, MI surgery still faces challenges in being widely adopted, particularly in low-resource areas where conventional MS is more common due to its lower costs and the availability of experienced surgeons. While the long-term benefits of MI thoracotomy are encouraging, more studies are needed to evaluate issues like the recurrence of defects, irregular heart rhythms, and whether it is cost-effective over time [16-18]. Therefore, the primary objective of this study was to compare the early postoperative outcomes, including pain levels, recovery time, and complication rates, between MI thoracotomy and conventional MS for ASD closure. Additionally, the study seeks to assess the potential advantages of MI thoracotomy in terms of cosmetic outcomes, hospital stay, and the need for analgesics. By comparing these approaches, the study aims to provide a comprehensive understanding that can guide clinical decision-making, optimize treatment strategies, and improve patient outcomes.

## Materials and methods

This comparative study aimed to evaluate the clinical outcomes of two surgical techniques for ASD closure: MI thoracotomy and conventional MS. Conducted between February 2021 and August 2024 at the Department of Cardiac Surgery, Bangabandhu Sheikh Mujib Medical University in Dhaka, Bangladesh, the study included 50 patients diagnosed with ASD. Fifteen patients were assigned to Group A, undergoing MI thoracotomy, while 35 patients in Group B underwent conventional MS. The Institutional Ethical Committee of Bangabandhu Sheikh Mujib Medical University approved the study (approval number: BSMMU/2021/6807).

Inclusion and exclusion criteria

Patients aged over 16 years with a single ASD were included in the study. Patients requiring additional interventions, such as the Maze procedure, mitral valve annuloplasty, or tricuspid valve annuloplasty, were excluded.

Power analysis of sample size

A power analysis was conducted using G*Power version 3.1 (Heinrich-Heine-Universität Düsseldorf, Düsseldorf, Germany) to determine the required sample size for the study. Previous studies have highlighted significant differences in postoperative recovery outcomes, such as pain levels, hospital stays, and complication rates, between the two surgical techniques. Based on this information, an effect size of 0.8 was chosen. The analysis was conducted with a significance level (α) of 0.05 and a statistical power (1-β) of 0.8, ensuring the study could reliably detect meaningful differences between the groups. This calculation indicated a minimum sample size of 44 patients. The final sample size was increased to 50 patients to account for potential data loss or dropouts.

Data collection

Clinical records were retrospectively reviewed to gather data on preoperative, intraoperative, and postoperative variables. Key demographic variables, including age, gender, comorbidities, and preoperative ASD diameters, were analyzed. Operative factors such as cardiopulmonary bypass duration and cross-clamp time were also evaluated. Postoperative data included complications, residual shunts, extubation time, inotropic support, hospital stay duration, and mortality. Pain management was assessed using the visual analog scale based on analgesic use during the first two postoperative days. The timing of patient discharge was also recorded.

Surgical techniques

Minimally Invasive Thoracotomy

Patients in Group A were administered standard anesthesia, with intubation performed using a double-lumen endotracheal tube to allow one-lung ventilation. Anticoagulation was achieved using a half-dose of heparin (2 mg/kg) to ensure adequate blood thinning. Transesophageal echocardiography (TEE) was performed after percutaneous cannulation of the right internal jugular vein using a 17- or 19-F arterial cannula. A small 2-cm incision was made in the right groin for femoral artery and vein cannulation, guided by Doppler ultrasound and performed using the Seldinger technique, with guidewire placement confirmed through TEE.

The patients were positioned at a 45-degree left lateral decubitus angle, and a 4-6 cm right anterolateral mini-thoracotomy was performed in the fourth intercostal space. A soft tissue retractor was used instead of rib-spreading instruments to minimize trauma. To provide visualization, a 5-mm high-definition camera (Karl Storz, Germany) was inserted through the fourth intercostal space. A transthoracic clamp (Estech Cobra Flex, USA) was applied via a cardioplegia cannula placed in the ascending aorta through the third intercostal space. Carbon dioxide insufflation was used throughout the procedure to prevent air embolism. Fine instruments, including 3-mm surgical scissors and forceps (Medtronic, USA), were employed to reduce tissue trauma. Continuous monitoring with TEE ensured proper shunt closure and helped detect potential air embolisms. If complications occurred, such as significant bleeding or inadequate visualization, the procedure was converted to a full sternotomy to ensure the patient’s safety.

Conventional Median Sternotomy

Patients in Group B underwent surgery through a midline incision under standard anesthesia. Aortic and bicaval cannulation was performed to enable ASD repair using cardiopulmonary bypass. During the procedure, warm cardioplegia was administered to protect the heart muscle while the cross-clamp was in place. The ASD was accessed through a right atriotomy and closed directly if a patch was not required. The atrium was closed once the repair was completed, and cardiopulmonary bypass was discontinued. Standard surgical tools, such as an aortic clamp (Geister, Germany) and retractors, were used throughout the procedure. If complications like bleeding or poor visualization arose, adjustments were made, such as administering additional cardioplegia or extending cross-clamp time to ensure the safety and effectiveness of the surgery.

Anesthetic protocols and intraoperative management

All patients received general anesthesia following standardized protocols. Premedication consisted of midazolam (0.03-0.05 mg/kg), followed by induction with propofol (1-2 mg/kg), fentanyl (2-4 mcg/kg), and rocuronium (0.6 mg/kg) to facilitate intubation. Anesthesia was maintained with isoflurane (0.8-1.2%) and intermittent boluses of fentanyl. Patients in Group A were intubated using a double-lumen endotracheal tube for one-lung ventilation, while standard intubation was used for patients in Group B.

During surgery, fluid balance was managed carefully with crystalloids (e.g., Ringer’s lactate or normal saline) and colloids to maintain hemodynamic stability. Vasopressors like norepinephrine (0.05-0.1 mcg/kg/min) were administered if required to maintain blood pressure. Anticoagulation was achieved with heparin (3 mg/kg) during cardiopulmonary bypass, and protamine sulfate was used to reverse its effects once the procedure was completed. Pain management after surgery included intravenous morphine (0.1 mg/kg) or patient-controlled analgesia to ensure patient comfort.

Data analysis

Data were analyzed using SPSS Statistics version 26.0 (IBM Corp. Released 2019. IBM SPSS Statistics for Windows, Version 26.0. Armonk, NY: IBM Corp.). Continuous variables were expressed as mean ± standard deviation (SD), while categorical variables were presented as frequencies and percentages. The Mann-Whitney U and independent samples t-test were used to compare continuous variables. Fisher’s exact test was applied to categorical data when chi-square assumptions were unmet. A p-value of less than 0.05 was considered statistically significant.

## Results

In Table 1, the demographic characteristics of the two groups are summarized. Group A had an average age of 31.77 ± 2.64 years, compared to 36.33 ± 12.61 years in Group B (p = 0.624), indicating no significant age difference between the groups. The body mass index (BMI) was 20.6 ± 0.7 in Group A and 21.7 ± 0.8 in Group B (p = 0.866), suggesting similar BMI distributions. The ASD size averaged 13.36 ± 3.13 mm in Group A and 17.21 ± 4.33 mm in Group B, with a p-value of 0.174, which was not statistically significant. Gender distribution was also comparable: Group A included six males (40%) and nine females (60%), while Group B comprised 12 males (34.29%) and 23 females (65.71%) (p = 0.124).

**Table 1:** Demographical characteristics of the study population in both groups SD: standard deviation, BMI: body mass index, ASD: atrial septal defect * Fisher's exact test

Variables	Group A mean ± SD	Group B mean ± SD	Test value	p-value
Age (in years)	31.77 ± 2.64	36.33 ± 12.61	-0.5	0.624
BMI (kg/m2)	20.6 ± 0.7	21.7 ± 0.8	-0.18	0.866
ASD size (mm)	13.36 ± 3.13	17.21 ± 4.33	-1.47	0.174
Gender			1.00*	0.12
Male (N, %)	6 (40%)	12 (34.29%)	-	-
Female (N, %)	9 (60%)	23 (65.71%)	-	-

In Table 2, the two groups observed significant differences between specific intraoperative parameters. The total operation time was significantly longer in Group A (157 ± 9.8 minutes) compared to Group B (123 ± 12.3 minutes), with a statistically significant difference (p < 0.001). This extended duration in Group A could be attributed to the complexity of the MI thoracotomy, which involves smaller incisions, precise instrument handling, and additional steps such as femoral cannulation and carbon dioxide insufflation. Intraoperative temperatures were also significantly lower in Group A. Rectal temperatures averaged 28.9 ± 1.1°C in Group A versus 33.2 ± 0.9°C in Group B, while nasopharyngeal temperatures were 26.2 ± 1.7°C in Group A compared to 31.2 ± 1.9°C in Group B (p < 0.001 for both). Cardiopulmonary bypass time (51.64 ± 13.5 minutes in Group A vs. 42.43 ± 8.1 minutes in Group B, p = 0.621) and cross-clamp time (28.67 ± 7.3 minutes in Group A vs. 22.9 ± 6.6 minutes in Group B, p = 0.492) were not significantly different between the groups. Although the volume of intraoperative blood product infusion was higher in Group B (129.7 ± 309.2 mL) than in Group A (15.8 ± 56.3 mL), this difference did not reach statistical significance (p = 0.139).

**Table 2:** Intraoperative variables of the study population in both groups A p-value <0.05 was considered statistically significant SD: standard deviation, Group A: minimally invasive thoracotomy, Group B: conventional median sternotomy

Variables	Group A mean ± SD	Group B mean ± SD	t-test value	p-value
Cardiopulmonary bypass time (min)	51.64 ± 13.5	42.43 ± 8.1	1.21	0.621
Cross clamp time (min)	28.67 ± 7.3	22.9 ± 6.6	1.25	0.492
Total operation time (min)	157 ± 9.8	123 ± 12.3	6.55	<0.001
Lowest rectal temperature (^o^C)	28.9 ± 1.1	33.2 ± 0.9	-11.9	<0.001
Lowest nasopharyngeal temperature (^o^C)	26.2 ± 1.7	31.2 ± 1.9	-9.7	<0.001
Intraoperatively infused blood products (mL)	15.8 ± 56.3	129.7 ± 309.2	-1.5	0.139

**Figure 1:**
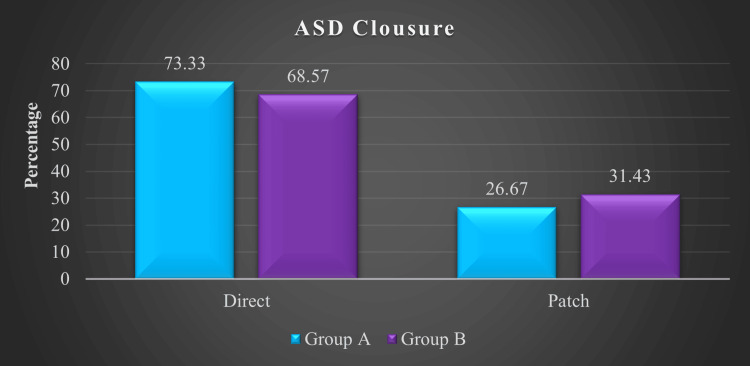
Type of ASD closure of the patients ASD: atrial septal defect, Group A: minimally invasive thoracotomy, Group B: conventional median sternotomy

Figure 1 illustrates the methods used for ASD closure. In Group A, 11 (73.3%) patients underwent direct ASD closure, while four (26.67%) patients required patch closure. In Group B, 24 (68.57%) patients had direct closure, and 11 (31.43%) patients underwent patch closure.

In Table 3, Group A demonstrated significantly higher postoperative drainage within the first 24 hours (288 ± 78.57 mL) than Group B (172.02 ± 92.66 mL, p = 0.048). Mechanical ventilation time was lower in Group A (5.89 ± 1.07 hours) than in Group B (6.9 ± 1.1 hours); however, this difference was not statistically significant (p = 0.219). Postoperative blood requirements were higher in Group B (371.2 ± 1,198.1 mL) compared to Group A (187.7 ± 549.8 mL), but this difference was not significant (p = 0.917). Similarly, the length of ICU stay (1.1 ± 0.06 days in Group A vs. 1.52 ± 0.3 days in Group B, p = 0.192) and total hospital stay (4.98 ± 1.01 days in Group A vs. 5.98 ± 1.02 days in Group B, p = 0.638) were comparable between the groups. A significantly higher proportion of patients in Group B required inotropic support (nine patients, 25.71%) compared to Group A (two patients, 13.33%) (p = 0.011). No residual defects were observed in either group.

**Table 3:** Postoperative variables of both groups A p-value <0.05 was considered statistically significant, * Fisher's exact test SD: standard deviation, ICU: intensive care unit, Group A: minimally invasive thoracotomy, Group B: conventional median sternotomy

Variables	Group A mean ± SD	Group B mean ± SD	Test value	p-value
Postoperative drainage for first 24 hours (ml)	288 ± 78.57	172.02 ± 92.66	2.02	0.048
Mechanical ventilation time (hour)	5.89 ± 1.07	6.9 ± 1.1	-1.3	0.219
Postoperatively infused total blood products (mL)	187.7 ± 549.8	371.2 ± 1,198.1	-0.10	0.917
ICU (day)	1.1 ± 0.06	1.52 ± 0.3	-1.5	0.192
Hospital length of stay (day)	4.98 ± 1.01	5.98 ± 1.02	-0.47	0.638
Blood product requirement (N, %)	1 (6.67%)	4 (11.43%)	3.52*	0.392
Inotropic support (N, %)	2 (13.33%)	9 (25.71%)	4.29*	0.011

Regarding postoperative complications, no significant differences were observed between the groups. Reoperation for bleeding was required in one patient (2.86%) in Group B, with no cases reported in Group A (p = 0.998). Neurological complications and wound infections were absent in both groups. Atrial fibrillation occurred in one patient (6.67%) in Group A and three patients (8.57%) in Group B (p = 0.187). Arrhythmias were observed in one patient (6.67%) in Group A and two patients (5.71%) in Group B (p = 0.091). The incidence of atelectasis was higher in Group A (two patients, 13.33%) compared to Group B (one patient, 2.86%), but this difference was not statistically significant (p = 0.153). Meanwhile, no conversions to sternotomy or mortality were reported in either group (Table 4).

**Table 4:** Postoperative complications of both groups N/A: not applicable, Group A: minimally invasive thoracotomy, Group B: conventional median sternotomy

Complications	Group A N (%)	Group B N (%)	Fisher's exact test value	p-value
Reoperation for bleeding	0 (0%)	1 (2.86%)	1.0	0.998
Atrial fibrillation	1 (6.67%)	3 (8.57%)	0.77	0.187
Arrhythmia	1 (6.67%)	2 (5.71%)	0.89	0.091
Atelectasis	2 (13.33%)	1 (2.86%)	0.26	0.153
Seroma	3 (20%)	0 (0%)	4.0	N/A

**Figure 2:**
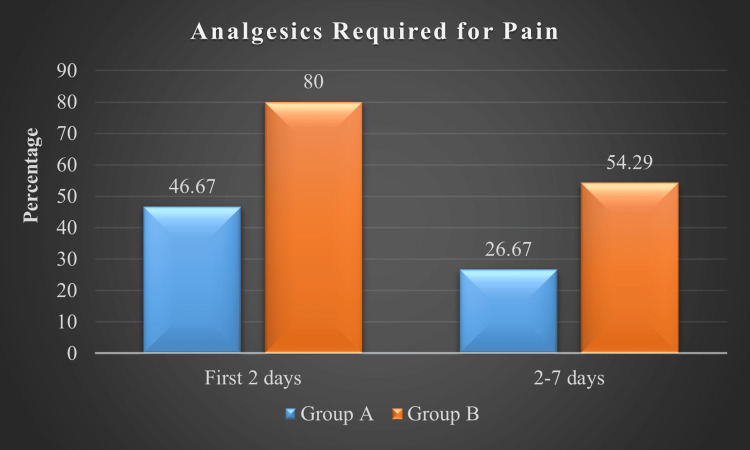
Postoperative pain requiring analgesics Group A: minimally invasive thoracotomy, Group B: conventional median sternotomy

Figure 2 illustrates the differences in pain management requirements between the groups. During the first two postoperative days, seven patients (46.67%) in Group A required analgesics compared to 28 (80%) in Group B. From days 2 to 6, analgesic use decreased to four patients (26.67%) in Group A and 19 (54.29%) in Group B. These results indicate that patients in Group B experienced higher postoperative pain levels requiring medication throughout the recovery period.

## Discussion

ASD repair can be safely and effectively performed using various surgical techniques, including conventional MS, cardioplegia, and cardiopulmonary bypass. The development of MI surgical methods, characterized by smaller incisions, has introduced new possibilities for managing this condition and improving patient care [19]. Extensive literature supports the efficacy of ASD closure using both MI and conventional surgical techniques. ASD accounts for 10-15% of all CHDs and is the most common congenital heart defect in adults. Its prevalence is twice as high in women as in men [20]. Consistent with these findings, the female-to-male ratio in our cohort was 60% to 40% in the MI thoracotomy group and 65.71% to 34.29% in the conventional MS group.

Prior studies, such as Chu et al. (2014), reported that cardiopulmonary bypass and total operation durations were significantly shorter in the conventional MS group. However, the two groups observed no differences in drainage volumes [21]. Variations in drainage volumes across studies suggest that lower blood loss and reduced transfusion requirements are associated with better hemodynamic stability and fewer transfusion-related complications. In our cohort, no statistically significant differences between the groups were observed in drainage volumes or transfusion requirements. Additionally, the durations of aortic cross-clamping and cardiopulmonary bypass were similar in the conventional MS and MI thoracotomy groups [21]. Complication rates were also comparable across the groups. Gil-Jaurena et al. (2011) reported a 1-2% risk of phrenic nerve and sinus node damage; however, no such complications occurred in our cohort [22]. Furthermore, no deaths were observed, with mortality rates aligning closely with those reported in the literature [23,24]. Middle- and late-term complications, such as atrial arrhythmias, pericardial effusion, tamponade, and thrombus formation, have been reported in the literature. Notably, the most common complication in our study was anemia requiring transfusion. While cardiac tamponade did not occur, atrial arrhythmias were observed in two patients (5.71%) in the conventional MS group and one patient (6.67%) in the MI thoracotomy group. Respiratory complications were rare, with atelectasis being the most common. Atelectasis was observed in 13.33% of patients in the MI thoracotomy group (two cases) and 2.86% in the conventional MS group (one case). No cases of pneumonia, pneumothorax, or pleural effusion were noted.

Yaliniz et al. (2015) reported shorter intubation durations and reduced transfusion needs in the MI thoracotomy group; however, our study did not find significant differences in these parameters [23]. One of the main advantages of MI cardiac procedures is the reduced length of hospital stays. These benefits, including quicker mobilization, psychological relief, and smaller, more cosmetically favorable incisions, make these techniques more appealing. In our study, the average duration of intensive care and hospital stays was 4.98 ± 1.01 days, which supports these advantages. Furthermore, MI procedures have been associated with better thoracic stability, reduced postoperative pain, and earlier mobilization than conventional MS [24]. Pain management, assessed over the first two postoperative days and throughout the hospital stay, showed a notable reduction in analgesic use. This outcome was likely attributable to early mobilization, routine pain catheter use in MI thoracotomy patients, and expedited psychological recovery.

The success of MI techniques in cardiac surgery has also prompted increased attention to the cosmetic outcomes associated with smaller incisions [25,26]. Massetti et al. (1999) studied patients undergoing MI surgery. They found that perceptions of surgical scars, their impact on daily life, and cosmetic satisfaction were significantly higher among patients treated with MI techniques than with conventional methods and reduced morbidity [27]. In our study, patients in the MI thoracotomy group experienced significantly better cosmetic outcomes than those in the conventional MS group. MS scars, particularly in women, can lead to significant sociological and psychological challenges. Considering the excellent cosmetic results, consistent surgical success, and high patient satisfaction, MI techniques may represent the optimal approach, particularly for young female patients [28].

Limitations of the study

One of the key strengths of this study was its comparative design, which allowed for a direct analysis of two commonly used surgical techniques for ASD closure. Comparing MI thoracotomy and conventional MS provided valuable insights into their relative benefits and drawbacks. Additionally, various outcome measures, such as pain levels, recovery times, and complications, offered a comprehensive understanding of early postoperative outcomes for both approaches.

However, the study had some limitations. The relatively small sample size (n = 50) reduced its statistical power, which may have affected the reliability of the results. A larger sample size would have allowed for the detection of more minor differences between the groups and improved the generalizability of the findings. The retrospective design, which relied on existing medical records, introduced the possibility of biases, such as selection bias or missing data. A prospective, randomized trial would have provided more robust evidence by minimizing these limitations.

Another limitation was the lack of long-term follow-up data, which prevented an assessment of the durability of surgical outcomes and potential late complications. Future studies with extended follow-up periods would be needed to better understand both surgical techniques' long-term effectiveness and safety. Additionally, this study was conducted at a single institution, which may have limited the applicability of the findings to other settings with different patient populations or surgical practices. A multicenter study involving a more diverse patient group would have enhanced the generalizability and confirmed the results in broader clinical settings.

## Conclusions

MI thoracotomy demonstrates significant advantages over conventional MS for ASD closure, including reduced postoperative pain, quicker recovery, shorter hospital stays, and improved cosmetic outcomes. Both techniques show comparable efficacy in intraoperative and early postoperative outcomes; however, MI thoracotomy is associated with fewer complications and less reliance on analgesics. These findings highlight the potential of MI thoracotomy to become the preferred approach, particularly for younger and female patients, due to its less invasive nature and patient-centered benefits.

Notably, the long-term implications of MI thoracotomy, such as its impact on quality of life, recurrence rates, and overall patient satisfaction, warrant further investigation. Large-scale studies with extended follow-up periods are essential to confirm these benefits and ensure the generalizability of these findings. Such research will provide deeper insights into the role of MI thoracotomy in routine clinical practice and support evidence-based decision-making for ASD closure.
